# Diverse Stakeholders of Tumor Metabolism: An Appraisal of the Emerging Approach of Multifaceted Metabolic Targeting by 3-Bromopyruvate

**DOI:** 10.3389/fphar.2019.00728

**Published:** 2019-07-04

**Authors:** Saveg Yadav, Shrish Kumar Pandey, Yugal Goel, Mithlesh Kumar Temre, Sukh Mahendra Singh

**Affiliations:** School of Biotechnology, Institute of Science, Banaras Hindu University, Varanasi, India

**Keywords:** tumor metabolism, tumor microenvironment, metabolic inhibitors, 3-bromopyruvate, organ homeostasis

## Abstract

Malignant cells possess a unique metabolic machinery to endure unobstructed cell survival. It comprises several levels of metabolic networking consisting of 1) upregulated expression of membrane-associated transporter proteins, facilitating unhindered uptake of substrates; 2) upregulated metabolic pathways for efficient substrate utilization; 3) pH and redox homeostasis, conducive for driving metabolism; 4) tumor metabolism-dependent reconstitution of tumor growth promoting the external environment; 5) upregulated expression of receptors and signaling mediators; and 6) distinctive genetic and regulatory makeup to generate and sustain rearranged metabolism. This feat is achieved by a “battery of molecular patrons,” which acts in a highly cohesive and mutually coordinated manner to bestow immortality to neoplastic cells. Consequently, it is necessary to develop a multitargeted therapeutic approach to achieve a formidable inhibition of the diverse arrays of tumor metabolism. Among the emerging agents capable of such multifaceted targeting of tumor metabolism, an alkylating agent designated as 3-bromopyruvate (3-BP) has gained immense research focus because of its broad spectrum and specific antineoplastic action. Inhibitory effects of 3-BP are imparted on a variety of metabolic target molecules, including transporters, metabolic enzymes, and several other crucial stakeholders of tumor metabolism. Moreover, 3-BP ushers a reconstitution of the tumor microenvironment, a reversal of tumor acidosis, and recuperative action on vital organs and systems of the tumor-bearing host. Studies have been conducted to identify targets of 3-BP and its derivatives and characterization of target binding for further optimization. This review presents a brief and comprehensive discussion about the current state of knowledge concerning various aspects of tumor metabolism and explores the prospects of 3-BP as a safe and effective antineoplastic agent.

## Introduction

Oncogenic transformation is associated with a massive metabolic reprogramming in neoplastic cells, which bestows unmatched self-sufficiency of biosynthetic, bioenergetic, and redox homeostasis (Tarrado-Castellarnau et al., 2016; Costa and Frezza, 2017). The reorganized metabolism leads to the generation of unique intrinsic and extrinsic environments conducive for an unhindered neoplastic transformation, accelerated tumor progression, the evolution of chemoresistance, invasion, and metastasis (Sánchez-García, 2009; Tao et al., 2014). Thus, tumor metabolism is recognized as an emerging hallmark of cancer (Hanahan and Weinberg, 2011; Pavlova and Thompson, 2016). The importance of unique metabolic characteristics of cancer cells was aptly recognized by Nobel laureate Sir Otto Heinrich Warburg, who described that neoplastic cells predominantly acquire energy through the fermentation of glucose to lactate irrespective of the presence or absence of oxygen and functional mitochondrial machinery (Warburg, 1956). This phenomenon of “aerobic glycolysis” is also denoted as the “Warburg effect” (Hanahan and Weinberg, 2011). Despite the ongoing debate regarding the universal applicability of the Warburg hypothesis on neoplastic cells (Hsu and Sabatini, 2008; Xu et al., 2015), the last decade has witnessed a vast resurgence of research to recognize tumor metabolism as the central driving force underlying the manifestation of the oncogenic phenotype of neoplastic cells (Kroemer and Pouyssegur, 2008; Liberti and Locasale, 2016). Interestingly, tumor cells display a tremendous heterogeneity concerning the execution of the metabolic hallmarks, depending on a variety of internal and external regulatory factors (Marusyk and Polyak, 2010; Polyak, 2011; Dagogo-Jack and Shaw, 2018). Moreover, metabolic reprogramming of neoplastic cells is accompanied by an equally matching utilization and dissemination machinery, mainly composed of the rewired metabolic pathways (Cantor and Sabatini, 2012; Quail and Joyce, 2013; Wang et al., 2017).

**Figure 1:**
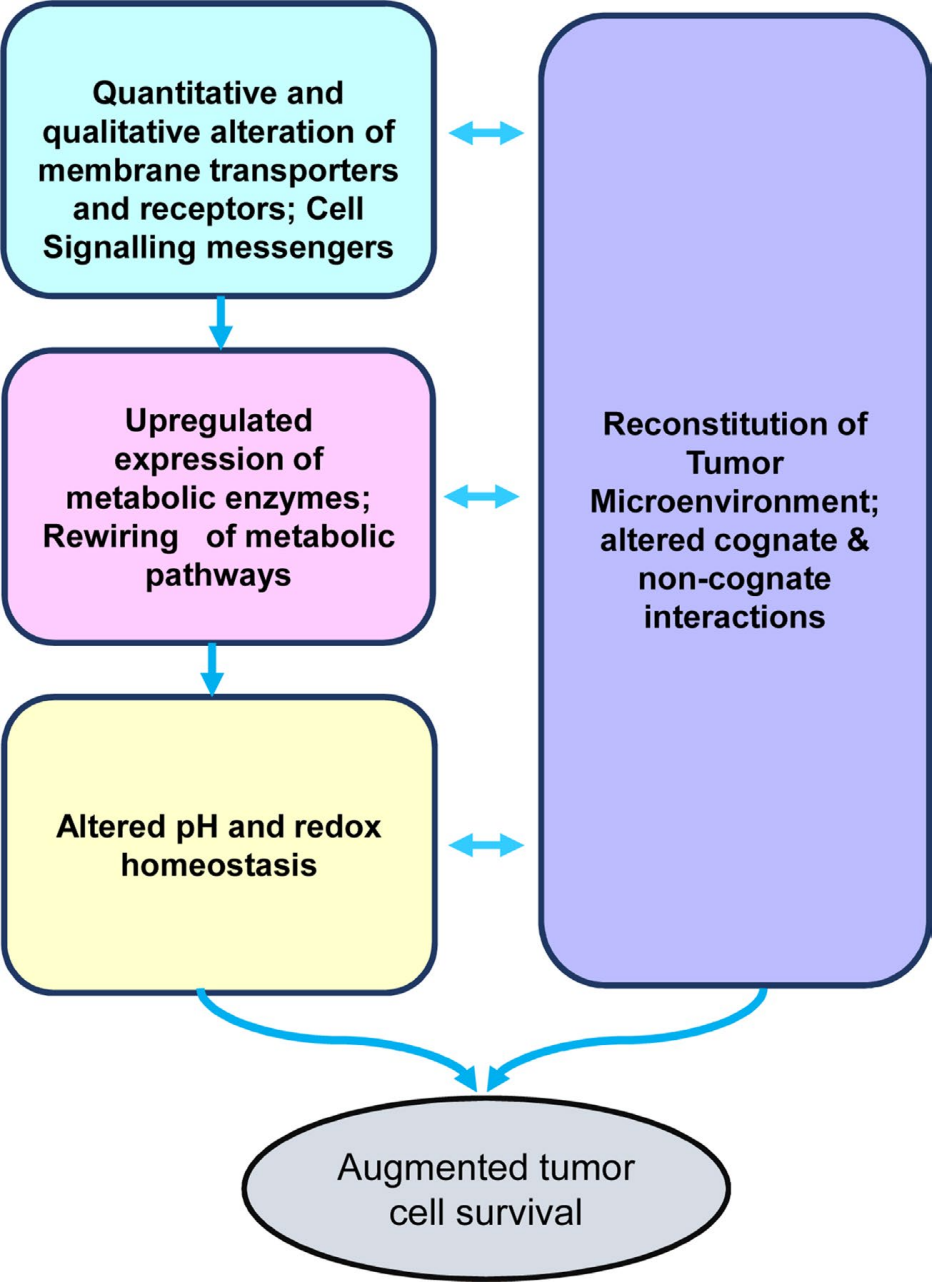
Stakeholders of tumor metabolism. The diagram depicts the primary stakeholders of the tumor metabolism, namely, nutrient uptake mechanisms, rewired metabolic pathways, altered pH homeostasis, and reconstituted tumor microenvironment, which collectively lead to the creation of a protumor survival scenario conducive for rapid tumor progression.

As depicted in **Figure 1**, the crucial aspects of tumor metabolism can be broadly categorized into the following major groups of functionalities: 1) quantitative and qualitative alterations of transporters’ repertoire for optimization of nutrient uptake; 2) acceleration of metabolic pathways caused by upregulated expression of the catalyzing enzymes and upstream signaling events; 3) altered pH and redox homeostasis, which facilitate the progression of metabolic pathways; and 4) metabolism-dependent alterations in the soluble, biophysical, and cellular components of the tumor microenvironment (TME), imparting diverse consequences on tumor progression. In the following sections of the review, we will discuss the above-mentioned crucial aspects of tumor metabolism and associated modulation of the TME. Furthermore, the review focuses on the promising ability of alkylating agent 3-bromopyruvate (3-BP) to circumvent these hallmarks of cancer metabolism, accompanying a discussion on issues related to its safety in antineoplastic therapeutic applications.

Although elegant reviews are addressing various aspects of the antineoplastic potential of 3-BP (Shoshan, 2012; Lis et al., 2016; Baghdadi, 2017), the novelty of the present review is a collation, on a single platform, of the updated and comprehensive information of multiple stakeholders of tumor metabolism. The particular focus of this review is on the repertoire of transporters involved in cancer metabolism *vis-à-vis* the ability of 3-BP to target most of the metabolic stakeholders. Furthermore, this review analyzes the available literature on the molecular characterization of the docking ability of 3-BP with some critical metabolic targets. Additionally, the review discusses the current literature addressing the recently reported effects of 3-BP on components of the TME and hematological homeostasis. This review also incorporates evidence addressing issues related to the safety of 3-BP on vital organs/systems, which was feebly discussed earlier.

## Optimization of Nutrient Uptake

### Glucose Metabolism

**Figure 2:**
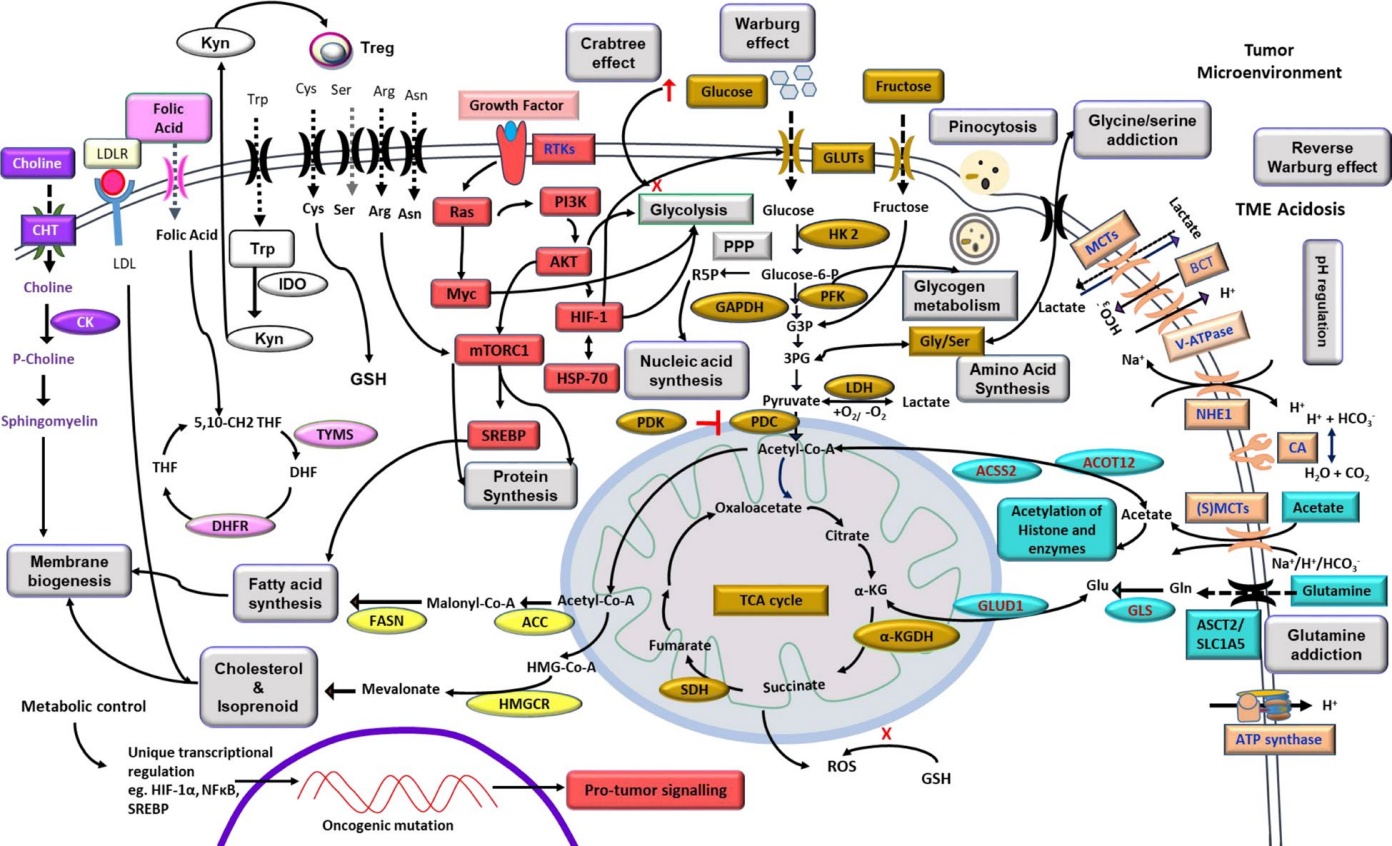
Highlights of tumor metabolism. The diagram depicts metabolic reprogramming in tumor cells with overexpression of transporters meant for nutrient uptake, pH regulation, and receptors for cytokines, hormones, and other ligands. Internal components include prominent metabolic pathways of bioenergetics and biosynthetic machinery, including carbohydrate and fatty acid metabolism, integration of metabolic networking for efficient utilization of substrates like glucose, fructose, lactate, acetate, amino acids, and precursor of membrane components. Signal transduction events indicate a crucial role of PI3K, HIF-1α, AKT, Ras, Myc, and mTOR downstream to receptor–ligand ligation with promoting consequences on metabolic pathways. The exterior of the membrane depicts a manifestation of the Warburg effect and modulation of the TME. Altered mitochondrial functions and its correlation to lipid metabolism, ROS generation, and glutamine assimilation are also depicted. The overall effect of such cross-talk of metabolic pathways resulted in the promotion of neoplastic cell survival. The diagram also indicates the metabolism-dependent regulation of gene expression. Carbohydrate metabolism is indicated by the golden color; cell signaling is indicated by the red color; alternative fuels and their cytoplasmic fates are indicated by the teal color; pH regulators are depicted by the orange color; choline metabolism is shown in purple color; enzymes of fatty acid and cholesterol synthesis are indicated by the yellow color; pink color represents folic acid metabolism; kynurenine and tryptophan pathways are depicted by the black and white boxes; amino acid transporters are shown in black; and major phenomena are indicated in boxes of gray color. Abbreviations: αKG, α-ketoglutarate; 3PG, 3phosphoglycerate; ACC, acetyl-CoA carboxylase; ACSS2, acyl-coenzyme A synthetase short-chain family member 2; ACOT12, acyl-CoA thioesterase 12; SLC1A5, neutral amino acid transporter B(0)/solute carrier family 1 member 5; CK, choline kinase; CHT, choline transporter; FASN, fatty acid synthase; GAPDH, glyceraldehyde-3phosphate dehydrogenase; DHF, dihydrofolic acid; DHFR, dihydrofolate reductase; GLUD1, glutamate dehydrogenase 1; GLS, glutaminase; GLUT, glucose transporter; G3P, glucose-3-phosphate; HIF, hypoxia inducible factor; HK2, hexokinase 2; HMG-CoA, 3-hydroxy-3–methyl-glutaryl coenzyme A; HMGCR, HMG-CoA reductase; HSP-70, 70-kilodalton heat shock protein; IDO, indoleamine 2,3-dioxygenase; Kyn, kynurenine; LDH, lactate dehydrogenase; MCT, monocarboxylate transporter; mTOR, mechanistic target of rapamycin; PDC, pyruvate dehydrogenase complex; NHE1, Na^+^/H^+^ exchanger 1; PDK, pyruvate dehydrogenase kinase; PI3K, phosphoinositide 3-kinase; PFK, phosphofructokinase; PPP, pentose phosphate pathway; R5P, ribose-5phosphate; ROS, reactive oxygen species; Treg, regulatory T cells; RTK, receptor tyrosine kinase; THF, tetrahydrofolic acid; TYMS, thymidylate synthetase; (S)MCT, sodium-coupled monocarboxylate transporter; SDH, succinate dehydrogenase; SREBP, sterol regulatory element-binding protein.

Neoplastic cells display an elaborate and upregulated expression of a plethora of transporters on their cell surface mainly meant for the uptake of nutrients required to fuel the accelerated metabolic pathways (**Figure 2**). Sugar transporters are particularly crucial, considering their role in fueling glycolysis. Neoplastic cells display an upregulated expression of glucose transporters (GLUT) like GLUT-1 and GLUT-3, facilitating the uptake of a huge quantity of glucose (Douard and Ferraris, 2008; Hamanaka and Chandel, 2012). Nevertheless, fructose transporter, GLUT-5, is also reported to be highly upregulated in several types of neoplastic cells (Douard and Ferraris, 2008). Moreover, studies have demonstrated an upregulated expression of additional sugar transports belonging to the sodium-dependent glucose cotransporter (SGLT) family, such as SGLT-1 and SLC5A/SGLT (Scafoglio et al., 2015). The role of other glucose transporters, however, remains debatable concerning their ability to fuel the metabolic pathways in neoplastic cells. Sporadic studies have implicated transporters such as GLUT-8 and GLUT-12 in specific types of neoplastic cells (Barron et al., 2012; Mueckler and Thorens, 2013). However, GLUT-1 and GLUT-3 remain as the unequivocally recognized universal glucose transporters responsible for the uptake of large quantities of glucose required by the highly glycolytic malignant cells (Mueckler and Thorens, 2013; Yu et al., 2017b). Thus, therapies aimed to target the implicated GLUTs are envisaged as novel antineoplastic strategies to interfere with neoplastic cells’ bioenergetic and biosynthetic homeostasis (Hamanaka and Chandel, 2012; Labak et al., 2016). Hence, many inhibitors of sugar transporters are being explored for therapeutic efficacy. As depicted in **Table 1**, drugs like cytochalasin B, resveratrol, naringenin, phloretin, WZB117, and thiazolidinedione have been demonstrated to display an inhibitory action on glucose transporters through direct (competitive) or indirect (noncompetitive) mechanisms (Kapoor et al., 2016; Siebeneicher et al., 2016). Furthermore, SGLT inhibitors such as dapagliflozin, canagliflozin, and empagliflozin have been explored for antineoplastic effectiveness (Lin and Tseng, 2014; Scafoglio et al., 2015). Approaches using short hairpin RNA (shRNA) and small interfering RNA (siRNA) to target the expression of various GLUTs are demonstrated to hold promising antineoplastic potential (Shimanishi et al., 2013; Jian et al., 2015). However, tumor cell specificity of these inhibitors is debatable because many healthy cells also express high levels of GLUT-1 and GLUT-3 under certain physiological conditions (Krzeslak et al., 2012). Additional indirect strategies to interfere with the “sugar tooth” of cancer cells include the targeting of signaling pathways, which regulate GLUT expression, implicating regulators such as phosphoinositide 3-kinase (PI3K), mechanistic target of rapamycin (mTOR), c-Myc, and hypoxia inducible factor (HIF)-1α (Choi, 2017). Inhibitors of such signaling mediator systems have been identified (**Table 1**) and are under active evaluation for therapeutic potential (Logue and Morrison, 2012). Similarly, vascular endothelial growth factor (VEGF)-dependent regulation of GLUT expression carries potential as a targetable entity of carbohydrate metabolism (Choi, 2017). Additionally, strategies involving lowering the availability of dietary sugars to minimize their uptake by the neoplastic cells have also been explored (Hamanaka and Chandel, 2012; Vishvakarma et al., 2013). Optimization of such approaches will be of potential benefit in circumventing the glucose dependence of cancer metabolism.

**Table 1:** Inhibitors of tumor metabolism.

Class of molecules	Inhibitor/trial stage		References
	Preclinical	Clinical	
**a) Transporters**			
Glucose transporter (GLUT)	Cytochalasin B, naringenin, phloretin, WZB117, thiazolidinedione	Silybin (SIL) (Phase I), resveratrol (Phase II)	Popat et al., 2013; Siegel et al., 2014; Kapoor et al., 2016; Siebeneicher et al., 2016
Sodium-dependent glucose cotransporters (SGLTs)	Dapagliflozin, canagliflozin, and empagliflozin		Lin and Tseng, 2014
Monocarboxylate transporters (MCTs)	AZD3965 and α-cyano-4-hydroxycinnamic acid		Doherty and Cleveland, 2013; Kumar et al., 2013a; Polański et al., 2014
Na^+^/monocarboxylate transport (SMCT)	Ibuprofen, ketoprofen, and fenoprofen		Ganapathy et al., 2008
Amino acid transporter	2-Aminobicyclo-(2,2,1)-heptane-2-carboxylic acid		Imai et al., 2010; Huttunen et al., 2016
Carbonic anhydrase, HCO_3_^−^ transporter, Na^+^/H^+^ exchanger	KR-33028, acetazolamide, biphenylsulfonamides, brinzolamide, and dorzolamide	Wilex’s cG250 (Rencarex) (Phase I/II)	Morsy et al., 2009; Siebels et al., 2011; Pinard et al., 2013; Amith et al., 2016; Bayat Mokhtari et al., 2017
Vacoular ATPase (V-ATPase)	NiK12192, FR202126	Pantoprazole (Phase I)	Pérez-Sayáns et al., 2009; Vishvakarma and Singh, 2011; Brana et al., 2014
Glutamine transporter	L-γ-glutamyl-p-nitroanilide		Choi and Park, 2018
Fatty acid translocase (CD36)	shRNA		Watt et al., 2019
LDH receptor	shRNA		Gallagher et al., 2017
**b) Signaling messenger/transcription factors**			
c-Myc	Mycro3, Mycomycin-2, FBN-1503, 10058-F4		McKeown and Bradner, 2014
Sterol regulatory element-binding protein (SREBP)	Betulin, fatostatin, PF-429242, FGH10019		Hawkins et al., 2008; Kamisuki et al., 2011; Tang et al., 2011; Li et al., 2014b
mTOR		XL765 (Phase I/II), AZD8055 (Phase I/II), NK128/MLN0128 (Phase I/II), Everolimus (Phase II), ridaforolimus (Phase III)	Porta et al., 2014; Xie et al., 2016; Fenner et al., 2019
PI3K/AKT and mTOR pathways	NVP-BEZ235	Metformin (Phase III), miltefosine (Phase III)	Porta et al., 2014
**c) Metabolic enzymes**			
Hexokinase 2 (HK2)	3-BP, 2-Deoxy-D-glucose	Lonidamine (Phase II)	Oudard et al., 2003; Granchi and Minutolo, 2012; Gandham et al., 2015; Roberts and Miyamoto, 2015; Yadav et al., 2017b
Lactate dehydrogenase (LDH)	Sodium oxamate, FX-11, galloflavin	Gossypol (Phase II)	Baggstrom et al., 2011; Doherty and Cleveland, 2013; Zhao et al., 2015b
Glutaminase	BPTES, CB-839, compound 968		Hensley et al., 2013; Choi and Park, 2018
Glutamate dehydrogenase		Epigallocatechin-3-gallate (Phase I)	Hensley et al., 2013; Zhao et al., 2014
Acetyl-CoA synthetase II	AR-12/OSU-03012		Koselny et al., 2016
Adenosine triphosphate citrate lyase (ACLY)	Hydroxycitrate, radicicol, purpurone, MEDICA		Zu et al., 2012
Acetyl-CoA carboxylase (ACC)	MK-4074, ND-630, Soraphen-A		Beckers et al., 2007; Harriman et al., 2016; Kim et al., 2017
Fatty acid synthase (FASN)	Orlistat, cerulenin and its derivative C75	Epigallocatechin-3-gallate (Phase I)	Kant et al., 2014b; Zhang et al., 2016a; Zhang et al., 2016b
Succinate dehydrogenase (SDH)	α-tocopheryl succinate, mitochondrially targeted vitamin E succinate (MitoVES), 3-BP malonate, nitropropionic acid thenoyltrifluoroacetone, troglitazone, atpenin A5		Burstein et al., 2003; Kluckova et al., 2013
Fumarate hydratase	Pyrrolidinone analogs 1–3		Takeuchi et al., 2015
Isocitrate dehydrogenase	AG120 and AG221	Enasidenib (Phase I)	Boddu and Borthakur, 2017; Li et al., 2018
Glyceraldehyde 3-phosphate dehydrogenase (GAPDH)	Koningic acid, methylglyoxal, saframycin A, 3-BP		Ganapathy-Kanniappan et al., 2012; Liberti et al., 2017; Liberti et al., 2019
Arginine-depleting enzymes	Arginase, arginine decarboxylase,	Arginine deiminase	Patil et al., 2016; Riess et al., 2018
Depletion of amino acids	L-asparaginase		Esen et al., 2016
Indoleamine 2,3-dioxygenase	Navoximod	Epacadostat (Phase II), indoximod (Phase I),	Brochez et al., 2017; Kristeleit et al., 2017
Oxidative stress inducer		Elesclomol (STA-4783)	O’Day et al., 2013
Pyruvate dehydrogenase kinase		Dichloroacetate (Phase I)	Chu et al., 2015
Glutathione (GSH)		Phenethylisothiocyanate (Phase I), Imexon (amplimexon) (Phase II)	Barr et al., 2014; Yuan et al., 2016
Isocitrate dehydrogenase 1 (IDH1)		AG-120 (Phase I)	Stein et al., 2014
HMG-CoA reductase		Statins (Phase III), SWOG0919 (Phase II)	Advani et al., 2014; Lim et al., 2015; Lee et al., 2017b
**d) Other targets **			
Vascular endothelial growth factor (VEGF)		Bevacizumab (Phase II),sorafenib (Phase II), sunitinib (Phase II), pazopanib (Phase II)	Moreira et al., 2007; Lane et al., 2009; Li et al., 2014a; Chan et al., 2018; Sato et al., 2018; Sun et al., 2018; Beppu et al., 2019; Fenner et al., 2019

**Table 1** highlights the stage of preclinical and clinical trials of the indicated metabolic inhibitors. Some of these inhibitors yielded limited therapeutic success. The inhibitor of monocarboxylate transporter (MCT) lonidamine did not pass phase III clinical trial (Berruti et al., 2002). Likewise, failure is reported for oxidative stress inducer STA-4783 (Sborov et al., 2015). Similarly, mTOR inhibitors fetched limited success in clinical trials (Faes et al., 2017). Moreover, VEGF inhibitor bevacizumab and other such agents fetched limited success in clinical trials (Zirlik and Duyster, 2018). The possible reasons underlying the observed failure of clinical trials could be as follows: 1) lack of adequate basic and preclinical research foundation before translational application in cancer patients; 2) obtaining approval for the clinical trials is a time-consuming process, requiring liaison of basic researchers, clinicians, pharmacologists, and financial sponsors; 3) limitations regarding the bioavailability of inhibitors within the tumor milieu in adequate cytotoxic concentration; 4) toxicity and other side effects of inhibitors in clinical applications; and 5) limitations of knowledge regarding the metabolomics of human cancers to determine their susceptibility in an inhibitor-specific manner.

### Lipid Metabolism

Lipid metabolism is crucial for tumor cell survival, particularly concerning membrane biogenesis and cell signaling to sustain rapid cellular proliferation (Beloribi-Djefaflia et al., 2016). Moreover, levels of lipids like cholesterol, high-density lipoprotein, and low-density lipoprotein (LDL), and their metabolic by-products are significantly elevated in tumor-bearing hosts (Beloribi-Djefaflia et al., 2016). Lipid uptake of neoplastic cells is mediated *via* various modes. Passive diffusion of lipids is considered as one of the main routes through which fatty acids gain entry in neoplastic cells (Harjes et al., 2016). Accumulating experimental evidence has demonstrated that the neoplastic cells mostly produce their lipids by fatty acid synthase (FASN) catalyzed *de novo* fatty acid synthesis (Santos and Schulze, 2012). Orlistat, an inhibitor of FASN, has been demonstrated to circumvent tumor cell survival effectively (Kant et al., 2012, Kant et al., 2014b; Schcolnik-Cabrera et al., 2018). Nevertheless, many studies have also indicated lipolysis as an additional source of fatty acids (Zaidi et al., 2013; Zhao et al., 2017). Moreover, lipophagy is yet another alternative source of lipids, which is associated with oncogenic transformation and metastasis (Maan et al., 2018). Thus, neoplastic cells display altered “lipid metabolic network” to sustain their bioenergetic and biosynthetic processes (Maan et al., 2018). Neoplastic cells also display upregulated expression of a transmembrane fatty acid translocase (CD36), a scavenger receptor, which is responsible for fatty acid and protein uptake (Enciu et al., 2018). Hence, approaches to inhibit the transporter functions of CD36 (**Table 1**) can cause inhibition of both protein and lipid supply to cancer cells (Watt et al., 2019). Moreover, the expression of LDL receptor is highly upregulated in neoplastic cells of diverse etiologies, which are internalized after the ligation to the LDL (Furuya et al., 2016). The expression of LDL receptor is mainly regulated by a membrane-bound transcription factor designated as sterol regulatory element-binding protein (SREBP1) (Streicher et al., 1996). Additionally, SREBP has been shown to regulate several key processes of lipid metabolism, including the uptake of cholesterol, fatty acids, triglycerides, phospholipid, and Nicotinamide adenine dinucleotide phosphate (NADPH) (Guo et al., 2014). Furthermore, the upregulated lipid metabolism of cancer cells is dependent on various factors, including hypoxia, tumor acidosis, and upregulated SREBP1c *via* signaling of Ras, extracellular signal-regulated kinase1/2, Phosphatase and tensin homolog, PI3K, and Protein kinase B (PKB or AKT) (Santos and Schulze, 2012; Beloribi-Djefaflia et al., 2016). SREBP also activates adenosine triphosphate citrate lyase (ACLY), acetyl-CoA carboxylase (ACC), and FASN in neoplastic cells (Baenke et al., 2013; Guo et al., 2014). ACLY, in turn, catalyzes the conversion of citrate to acetyl-CoA, which is then converted to malonyl CoA by the action of ACC. The malonyl CoA serves as a substrate for FASN to produce fatty acids. The fatty acids thus generated serve as a major source of signaling proteins, membrane phospholipids, and production of acyl-CoA to be channelized into the tricarboxylic acid cycle (TCA) cycle (Baenke et al., 2013). The electrons released from the β-oxidation of lipids are utilized for the production of NADPH and flavin adenine dinucleotide (FADH2) for redox balancing and adenosine triphosphate (ATP) production (Santos and Schulze, 2012). Moreover, lipids are stored as lipid droplets in cancer cells, which render protective action in cellular adaptations during the stressful condition of nutrient depletion (Baenke et al., 2013). Reports also indicate a major contribution of lipid metabolism in the metastasis of neoplastic cells (Luo et al., 2017). Consequently, lipid metabolism appears to be a multifaceted “metabolic lifeline” of neoplastic cells, indicating a strong potential for one or more of these pathways as targets for therapeutic maneuvering. Furthermore, the therapeutic utility of targeting LDL receptor in pancreatic cancer cells has been demonstrated to hold a promising antineoplastic potential (Vasseur and Guillaumond, 2016). The approach of shRNA-mediated interference of LDL receptor expression was also shown to chemosensitize neoplastic cells (Gallagher et al., 2017) (**Table 1**).

### Alternative Nutrients

Neoplastic cells gain an advantage in their battle for resources by the modulation of their sole metabolic dependence on sugars (Cantor and Sabatini, 2012; DeBerardinis and Chandel, 2016) by utilizing other nutrients, which serve as substrates for driving various metabolic pathways. Such nutrients are collectively designated as “alternative fuels” (Keenan and Chi, 2015; Cairns and Mak, 2016). The alternative fuels of neoplastic cells include a variety of biomolecules such as lactate, acetate, glutamine, cysteine, alanine, and several proteins, which can be channeled into the metabolic pathways (Keenan and Chi, 2015; Sousa et al., 2016). In addition to the utility of alternative fuels in carbohydrate catabolism, they are also used in lipid, protein, and histone metabolism (Keenan and Chi, 2015). Moreover, glutamine contributes to the redox homeostasis of tumor cells (Vučetić et al., 2017; Choi and Park, 2018). Many neoplastic cells overexpress transporters for glutamine and other amino acids (Hensley et al., 2013; Zhao et al., 2015a; Lukey et al., 2017; Choi and Park, 2018). Additionally, transporters including MCT-1 (SLC16A1) and MCT-4 (SLC16A3) mediate the uptake of lactate and branched-chain keto acids (Kennedy and Dewhirst, 2010; Silva et al., 2017), SLC7A11 for cysteine (Huang et al., 2005), and ASCT2 for glutamine (Cormerais et al., 2018), facilitating the uptake of alternative fuels by cancer cells. Moreover, neoplastic cells display an upregulated expression of MCTs for lactate and acetate uptake (Birsoy et al., 2013). Acetate is also imported *via* the Na^+^/H^+^/HCO_3_^−^ transporter (Pandey et al., 2018). Additionally, acetate is endogenously generated in neoplastic cells (Pandey et al., 2018). The uptake of acetate by cancer cells is dependent on facilitated diffusion *via* aquaporins and through transporters like MCT-1/2 and Na^+^/monocarboxylate transport 1 (SMCT1) (Ferro et al., 2016; Pandey et al., 2018). Accumulating pieces of evidence demonstrate that neoplastic cells use acetate for the synthesis of acetyl-CoA and thus feed the TCA cycle and fatty acid synthesis (Lyssiotis and Cantley, 2014). Furthermore, tumor cells display upregulated expression of acetyl-CoA synthetase II (Lyssiotis and Cantley, 2014; Research, 2015). Acetate is also utilized for acetylation of histone of several metabolic enzymes. Moreover, acetate uptake by the tumor cells is linked to the uptake of Na^+^ and HCO_3_^−^, which can be utilized in the metabolic processes *via* the upregulated expression of SMCTs (Sterling and Casey, 2002). Lactate can be converted to pyruvate in neoplastic cells by the action of lactate dehydrogenase (LDH), showing upregulated expression in neoplastic cells (Miao et al., 2013). Additionally, lactate is implicated in the modulation of signaling events leading to activation of HIF-1α (Jiang, 2017). Nevertheless, lactate is also a source of carbon for cancer cells under normoxic conditions (Jiang, 2017). Furthermore, neoplastic cells display upregulated expression of the transporters of several non-glutamine and cysteine amino acids including serine, methionine, arginine, valine, leucine, asparagine, and glycine, collectively contributing to a higher uptake of amino acids by neoplastic cells (Schulze and Harris, 2012; Keenan and Chi, 2015; Hayase et al., 2017). Amino acid transporter LAT-1 (SLC7A5) and its chaperone CD98 also mediate uptake of neutral amino acids in cancer cells (Haase et al., 2007). Interestingly, whole proteins can be taken up *via* scavenger receptor CD36 and other processes including macropinocytosis, which facilitates the uptake of the lipids (Bonen et al., 2004; Ha et al., 2016). Signaling *via* Ras and Src facilitates the process of macropinocytosis by increasing vesicular transport (Commisso et al., 2013; Recouvreux and Commisso, 2017). Macropinocytosis is followed by a breakdown of engulfed molecules by the action of lysosomal enzymes (Recouvreux and Commisso, 2017) to be utilized in metabolic processes. Strategies are thus being designed to utilize the weakness of tumor cells for nutrient uptake for the import of anticancer drugs (Ha et al., 2016).

Approaches to inhibiting the transporters of alternative fuels have been experimented for designing antineoplastic strategies. MCT inhibitors α-CHC and AZD3965 have been demonstrated to inhibit tumor cell survival in a variety of neoplastic cells (Doherty and Cleveland, 2013; Kumar et al., 2013a, Kumar et al., 2013b; Polański et al., 2014; Curtis et al., 2017). Similarly, SMCT inhibitor ibuprofen and its derivatives show promising anticancer activity (Ganapathy et al., 2008). Moreover, inhibitors of other transporters like those of various amino acids and carbonic anhydrase display antineoplastic potential (Bhutia et al., 2015) (**Table 1**).

## Rewiring of Metabolic Pathways in Cancer

Commensurate to the repertoire of nutrient import mechanisms, neoplastic cells display highly upregulated metabolic pathways, particularly those implicating carbohydrates, amino acids, other alternative fuels, and lipids as substrates (Kroemer and Pouyssegur, 2008). These geared-up metabolic pathways are effectively maneuvered by an upregulated repertoire of metabolic enzymes (Lincet and Icard, 2015) and their regulatory elements (Mossmann et al., 2018). As tumor cells mainly depend on glycolysis for their ATP production, they display an augmented expression of mitochondrial membrane-associated hexokinase (HK), which catalyzes the conversion of glucose to glucose-6-phosphate, the first crucial step of glycolysis (Wilson, 2003; Mathupala et al., 2006). Additionally, the neoplastic cells display massive upregulation in the expression of other glycolytic enzymes, including phosphofructokinase, glyceraldehyde 3-phosphate dehydrogenase (GAPDH), and isoforms of pyruvate kinase (Ganapathy-Kanniappan and Geschwind, 2013). Interestingly, using a natural GAPDH inhibitor, koninginic acid, the group of Locasale (Liberti et al., 2017; Liberti et al., 2019) demonstrated that aerobic glycolysis (Warburg effect) and glucose metabolism are functionally distinct phenomena, a promising rationale for therapeutic targeting of the Warburg effect (Liberti et al., 2019). Besides the upregulated expression of transporters and enzymes mentioned above, HIF-1α contributes in the suppression of the mitochondrial OxPhos metabolism of glucose by inhibiting pyruvate dehydrogenase (PDH) *via* the upregulated expression of pyruvate dehydrogenase I (Kim et al., 2006; Singh et al., 2017). As stated earlier, neoplastic cells display an upregulated expression of LDH (Miao et al., 2013). LDH is also under the regulatory control of HIF-1α-associated signal transduction events (Luo et al., 2017). Additionally, glucose metabolism is regulated by other upstream signaling mediators, including p53, Ras, c-Myc, AKT, and mTOR (Hay, 2016). It is important to note that the accelerated metabolic machinery is regulated by oncogene activation in the normoxic condition itself, rendering tumor cells to manifest the Warburg effect. Thus, the HIF-1α-dependent upregulation of glycolysis in neoplastic cells is ancillary and manifested only under hypoxic conditions (Yu et al., 2017a). Nevertheless, the upregulated metabolism of glucose through glycolysis caters to the anabolic machinery for the synthesis of other biomolecules (Ganapathy-Kanniappan and Geschwind, 2013). Moreover, neoplastic cells are reported to display elevated channelization of glucose to the pentose phosphate pathway, which has a significant contribution in the biosynthesis and bioenergetic machinery (Patra and Hay, 2014). Additionally, the pentose phosphate pathway contributes to the generation of protons and, hence, in the maintenance of tumor acidosis (Zhang et al., 2017).

Although many earlier studies have suggested a truncated role of TCA cycle in carbohydrate metabolism, recent experimental evidence strongly indicates that even Krebs’ cycle is functionally operative in neoplastic cells and has a crucial role in the bioenergetics of carbohydrate, lipid, and aspartate metabolism, accompanying maintenance of redox homeostasis of cancer cells (Martínez-Reyes et al., 2016; Anderson et al., 2018; Sullivan et al., 2018). Moreover, it is overwhelmingly suggested that the TCA cycle facilitates neoplastic cells to utilize alternative fuels, such as glutamine and acetate (Keenan and Chi, 2015). Additionally, the TCA cycle plays a crucial role in cancer cells for anaplerotic reactions to support their biosynthetic machinery (Griss et al., 2015; Anderson et al., 2018). The metabolic signaling apparatus composed of Myc, HIF-1α, p53, and Ras plays a crucial regulatory role to reinforce the TCA cycle of neoplastic cells by triggering alterations in the expression of constituent enzymes such as succinate dehydrogenase (SDH), fumarase, and isocitrate dehydrogenase (IDH) (Raimundo et al., 2011). Moreover, the *de novo* synthesis of lipids utilizes citrate generated through the TCA cycle by the catalytic action of ACLY, ACC, and FASN, all of which are regulated by membrane-bound transcription factor SREBP (Madison, 2016). Furthermore, the electron transport chain (ETC) functions, in particular, respiratory complex I, are crucial for redox homeostasis in addition to its role in oxygen sensing and lipid and aspartate metabolism (Galkin et al., 2009; Vatrinet et al., 2015; Gui et al., 2016; Martínez-Reyes et al., 2016; Kurelac et al., 2019).

One carbon metabolism is also considered crucial for supporting processes such as nucleotide synthesis, methylation, and nicotinamide adenine dinucleotide (NAD^+^/NADH) generation (Newman and Maddocks, 2017; Rosenzweig et al., 2018). Neoplastic cells utilize molecules like folic acid, serine, and glycine to fuel the one-carbon metabolism (**Figure 2**) (Rosenzweig et al., 2018). Hence, antifolate agents have been considered for antineoplastic activity (Newman and Maddocks, 2017). It is also proposed that targeting one-carbon metabolism may render a promising contribution to the designing of novel anticancer therapeutic strategies (Newman and Maddocks, 2017).

## Unique pH Homeostasis of Neoplastic Cells: Generation of Tumor Acidosis

Tumor cells generate a massive amount of protons, which necessitates a tight regulation of the intracellular pH to prevent cytosolic acidification. Consequent pumping of these protons to the exterior causes the pH of the external milieu to reach the range of 6.5 to 6.9, designated as tumor acidosis (Damaghi et al., 2013). Interestingly, tumor acidosis also contributes to the manifestation of metabolic reprogramming of cancer cells (Peppicelli et al., 2017). Besides the significant contribution of accelerated metabolism in lowering pH, many other factors also contribute to the modulation of pH in cancer cells, which include, but is not limited to, hypoxia, hypercapnia, and a reduced diffusion of gases in the TME (Damaghi et al., 2013; Swietach et al., 2014; Damaghi et al., 2015). pH regulation results in a relative alkalinization of cytosol, accompanied by acidification of the external milieu (Chiche et al., 2010; Robey, 2012; Persi et al., 2018), having an up-regulatory action on tumor progression, metastasis, chemo-, and immuno-resistance (Riemann et al., 2016; Corbet and Feron, 2017; Huber et al., 2017; Persi et al., 2018). Accelerated glycolysis of neoplastic cells generates an enormous quantity of lactate, which is pumped to the exterior by MCTs (Swietach et al., 2014). Furthermore, recent reports highlight abnormal Golgi pH homeostasis in cancer cells, which is implicated in targeting carcinoembryonic antigen (Kokkonen et al., 2018). Moreover, glutamine metabolism is involved in the manifestation of tumor acidosis (Romero-Garcia et al., 2016). A hypoxic TME has also been demonstrated to be a critical trigger in regulating the expression of pH regulators, which play a crucial role in pH homeostasis and the manifestation of tumor acidosis (Miranda-Gonçalves et al., 2016). Nevertheless, the amount of glucose in the TME and transporters of nutritive molecules regulate the expression of various pH regulators (De Saedeleer et al., 2014; McGuire et al., 2016). Moreover, hypoxic conditions of the TME induce HIF-dependent cell signaling, which creates a glycolytic bias of glucose metabolism, leading to the high uptake of glucose, accelerated glycolysis, and production of lactate and H^+^ manifesting tumor acidosis (Petrova et al., 2018). HIF also promotes the expression of GLUTs and other nutrient transporters, fueling the upregulated glycolysis (Petrova et al., 2018). Furthermore, HIF supports pH homeostasis by promoting carbonic anhydrase (CA) IX expression (Iwasaki et al., 2015).

Besides MCTs, other membrane-associated pH regulators (**Figure 2**) include Na^+^/H^+^ exchanger (NHE), vacuolar ATPase (V-ATPase), CA, bicarbonate transporter (BCT), and ATP synthase (Damaghi et al., 2013; Swietach et al., 2014). Collectively, these pH regulators generate the characteristic “reverse pH gradient” across the plasma membrane of neoplastic cells, which is also recognized as a hallmark of the oncogenic transformation (Hanahan and Weinberg, 2011; Corbet and Feron, 2017). In addition to pH regulation, NHE, which belongs to the family of SLC cation/proton antiporters (CPAs), plays a crucial role in oncogenesis, tumor progression, and metastasis (Loo et al., 2012). NHE is responsible for exporting H^+^ with associated exchange of Na^+^ (Loo et al., 2012). Similar to other pH regulators, NHE expression is also dependent on signaling *via* PI3K, ras-related C3 botulinum toxin substrate 1 (Rac1), ERK1/2, and mitogen-activated protein kinase (MAPK) (Orlowski and Grinstein, 1997; Sartori et al., 1999; Vallés et al., 2015). In an interesting study using MCF-7 cells, it was demonstrated that malignant cells adapting to acidosis upregulate the expression of lysosomal protein LAMP2, which is translocated to the plasma membrane, rendering protection against acid-induced lysis (Damaghi et al., 2015). V-ATPases are yet another family of prominent pH regulators of neoplastic cells, which are known for a variety of normal cellular functions (Stransky et al., 2016). A wide spectrum of neoplastic cells is demonstrated to overexpress V-ATPase on their membrane (Stransky et al., 2016). Moreover, V-ATPase plays an indispensable role in pH homeostasis of neoplastic cells (Cotter et al., 2015). The expression of V-ATPase is under the regulatory control of signaling *via* Wnt/Notch and mTOR (Cotter et al., 2015; Stransky et al., 2016). In addition to its role in mediating export of H^+^ ion at the expense of the breakdown of ATP, V-ATPase facilitates autophagy, which is crucial in the biology of neoplastic cells (Stransky et al., 2016). Interestingly, recent studies have demonstrated the interaction between V-ATPase and microdomains of cholesterol in the manifestation of tumor metastasis (Stransky et al., 2016; Whitton et al., 2018). Thus, V-ATPase inhibitors are being explored for anticancer potential (**Table 1**). Furthermore, several reports emphasize the crucial role played by BCT in the regulation of pH in neoplastic cells (Gorbatenko et al., 2014). BCT belong to Na^+^/HCO_3_^−^ (SLC4 family) cotransporters and Cl^−^/HCO_3_^−^(SLC 26 family) exchangers. They display a modulated expression in neoplastic cells and mediate the process of pH regulation and several other functions of neoplastic cells (Gorbatenko et al., 2014). However, despite the promising potential of BCTs, only a few studies have been carried out to understand and evaluate their relative contribution in the maintenance of pH homeostasis of neoplastic cells (Kant et al., 2014a). CA II, CA IX, and CA XII subtypes are reported to play a crucial role in manifesting tumor acidosis (Mboge et al., 2018). CA IX is demonstrated to play a vital role in pH homeostasis of neoplastic cells (Benej et al., 2014). In addition to its role as a pH regulator, CA IX plays various other contributing roles in the biology of cancer cells, including epithelial–mesenchymal transition, reshaping other cognate cellular interactions in the TME, and altered chemosensitivity (Benej et al., 2014; Mboge et al., 2018). Among the anionic exchangers, anionic exchanger 2 has been well demonstrated for its role in pH regulation of neoplastic cells *via* its function to exchange chloride with HCO_3_^−^ (Shiozaki et al., 2018). It also plays a crucial role in tumor metabolism (Xu et al., 2009). Additionally, ectopic localization of F1/F0 ATP synthase from the mitochondrial membrane to cell surface plasma membrane in neoplastic cells is envisaged to play a role in tumor acidosis because it serves as a proton channel in addition to its participation in energy generation (Mowery and Pizzo, 2008). Nevertheless, the membrane of tumor cells displays an upregulated expression of numerous pH-sensing proteins, which include ovarian cancer G-protein-coupled receptor 1, G-protein-coupled receptor 4, T-cell death-associated gene 8, acid-sensitive ion channel, and transient receptor potential of channel vanilloid subfamily, which cooperate with pH regulators (Damaghi et al., 2013; Justus et al., 2013; Huber et al., 2017). Other pH-sensing proteins such as actin-depolymerizing factor/cofilin, talin, and guanine nucleotide exchange factors collectively cooperate to regulate microfilament remodeling, vital for epithelial-mesenchymal transition, tumor cell invasion, and metastasis (Damaghi et al., 2013). Thus, all of these pH regulators and pH-sensing proteins collectively act in a concerted manner to regulate prosurvival signaling, tumor progression, and metastasis (Kato et al., 2013; Riemann et al., 2016). Furthermore, acid-sensing ion channels, particularly acid-sensing ion channel 2, which are voltage-independent have been associated with tumor invasion and metastasis (Zhou et al., 2017). The transient receptor potential channel of vanilloid subfamily I (TRPVI) is a proton-sensitive channel associated with the regulation of the process of tumorigenesis (Bode et al., 2009). However, more studies will be required to decipher its role in pH regulation in cancer cells. In the view of the crucial role of pH-dependent metabolic reprogramming in cancer cells, various components of the pH regulatory machinery have been explored for therapeutic targeting. These include approaches such as alkalinization of the TME (Kato et al., 2013; Pilon-Thomas et al., 2016) and use of specific inhibitors of various pH regulators (Vishvakarma and Singh, 2010; Vishvakarma and Singh, 2011; Kuchuk et al., 2018). These approaches indicate that targeting pH homeostasis can result in a cytostatic action on tumor cell survival, proliferation, metastasis, and invasion (Swietach et al., 2014; Huber et al., 2017). Reversal of tumor acidosis also ushers augmented chemosensitivity, elimination of acidosis-induced immunosuppression, and retardation of angiogenesis (Justus et al., 2013; Thews et al., 2014; Huber et al., 2017; Lacroix et al., 2018).

## Metabolic Linking in the Tumor Microenvironment: A Platform for Redefining Cellular Relations

Although optimization of self-sufficiency is the main “motto” of neoplastic cells, external conditions become ultimately harsher, along with the progression of tumor, leading to a depleted supply of nutrients (DeBerardinis and Chandel, 2016). Moreover, tumor-infiltrating cells of the immune system compete for the available nutrients in the TME (Chang et al., 2015). Nutrient competition between tumor cells and tumor-infiltrating T lymphocytes has been elegantly demonstrated (Chang et al., 2015). However, being blessed with the Warburg phenomenon, neoplastic cells win the competition by comparatively higher uptake of glucose, leading to its depletion in the TME, thereby depriving the tumor-infiltrating cells of the immune system of essential glucose required for sustaining metabolism (Chang et al., 2015). Neoplastic cells evolve into a unique relationship with components of the TME, which could be symbiotic, parasitic, or competitive (Gatenby and Gillies, 2008; Lyssiotis and Kimmelman, 2017). Gradients of nutrients and gases in the TME create pockets of oxygenated, nutrient-rich, and depleted microniches (Lyssiotis and Kimmelman, 2017). Accordingly, both neoplastic and normal cells differentially adapt to these niches. Tumor cells optimize nutrient uptake by entering into a unique “metabolic symbiosis” operated between tumor cells themselves and with other normal cells in their vicinity (Nakajima and Van Houten, 2013). Thus, neoplastic cells can create a network of cognate and noncognate cellular interactions, among themselves, of “metabolic cross-feeding” to support nutritional uptake as an additional avenue. Lactate symbiosis is one of such well worked out examples, operational between aerobic and anaerobic tumor cells (Nakajima and Van Houten, 2013). Lactate produced by tumor cells is used as a nutrient not only by the OxPhos cancer cells but also by other cells of the TME, including mesenchymal stem cells, cancer-associated fibroblasts (CAFs), tumor-associated macrophages, and T lymphocytes (Allen et al., 2016; Romero-Garcia et al., 2016; Lyssiotis and Kimmelman, 2017). Furthermore, neoplastic cell-derived lactate has multiple effects on the metabolism of these stromal cells of the TME (Lyssiotis and Kimmelman, 2017), including M2 polarization of tumor-associated macrophages, which are protumorigenic, and inhibition of T-cell functions (Romero-Garcia et al., 2016; Yang and Zhang, 2017; Mu et al., 2018). Lactate is also reported to modulate redox and nitrogen balance in tumor cells (San-Millán and Brooks, 2017).

Availability of metabolites produced by normal cells in the TME also mediates the modulated metabolism of neoplastic cells (Lyssiotis and Kimmelman, 2017). Moreover, studies demonstrate that CAF produces lactate upon uptake of glucose, which in turn can be utilized by tumor cells (Lyssiotis and Kimmelman, 2017). Metabolic reprogramming of CAF leads to increased synthesis of glutamine (Lyssiotis and Kimmelman, 2017). Furthermore, increased uptake of glucose and tryptophan by tumor cells can deprive T cells of these nutrients, leading to an inhibition of their antitumor functions (Sukumar et al., 2015). Moreover, tryptophan metabolism of tumor cells produces kynurenine, which is reported to promote regulatory T cells to inhibit the functions of T helper (T_H_) cells and contribute to tumor growth promotion (Lyssiotis and Kimmelman, 2017). Moreover, a recent study strongly indicates the role of CD4^+^ T_H_ cells in gearing antitumor immune responses (Morales Del Valle et al., 2019). Hence, T_H_ cells are envisaged for anticancer therapeutic applications. Besides immune cells, adipocytes of the TME contribute to lipid homeostasis of neoplastic cells (Lyssiotis and Kimmelman, 2017). TME adipocytes produce fatty acids that are taken up by cancer cells to support their metabolism (Lyssiotis and Kimmelman, 2017). Tumor cells can also import mitochondria and exosomes containing metabolites (Lyssiotis and Kimmelman, 2017). Furthermore, the immune cells of the TME get suppressed because of nutrient deprivation caused by not only tumor cells but also other triggers derived from neoplastic cells and normal cells (Lyssiotis and Kimmelman, 2017). Indeed, arginine deprivation in the TME by cells of myeloid lineage is reported to be the cause of T-cell inhibition (Hanahan and Weinberg, 2011). Additionally, recent reports indicate the role of tumor acidosis in suppressing the activity of T effector cells and macrophages (Choi et al., 2013). Tumor acidosis is also conducive for tumor infiltration of immune cells, which, however, get suppressed or polarized to promote tumor progression (Choi et al., 2013). Furthermore, metabolites of the TME are capable of epigenetic modulations, such as histone acetylation, with consequences of modified genetic regulation of tumor cell metabolic plasticity (Etchegaray and Mostoslavsky, 2016). However, much still needs to be understood regarding the precise definition of cellular interactions at tumor–tumor and tumor–immune cells’ synapse, which has the potential for being explored for therapeutic reconstitution of the TME based on the interference of metabolic coupling between the constituent cells. Studies on 3D multicellular spheroids can be an important tool in understanding such dimensions concerning “metabolic coupling” operating in the TME (Nath and Devi, 2016).

## 3-Bromopyruvate Is Capable of Multifaceted Targeting of Tumor Metabolism and Constituents of the Tumor Microenvironment

**Figure 3:**
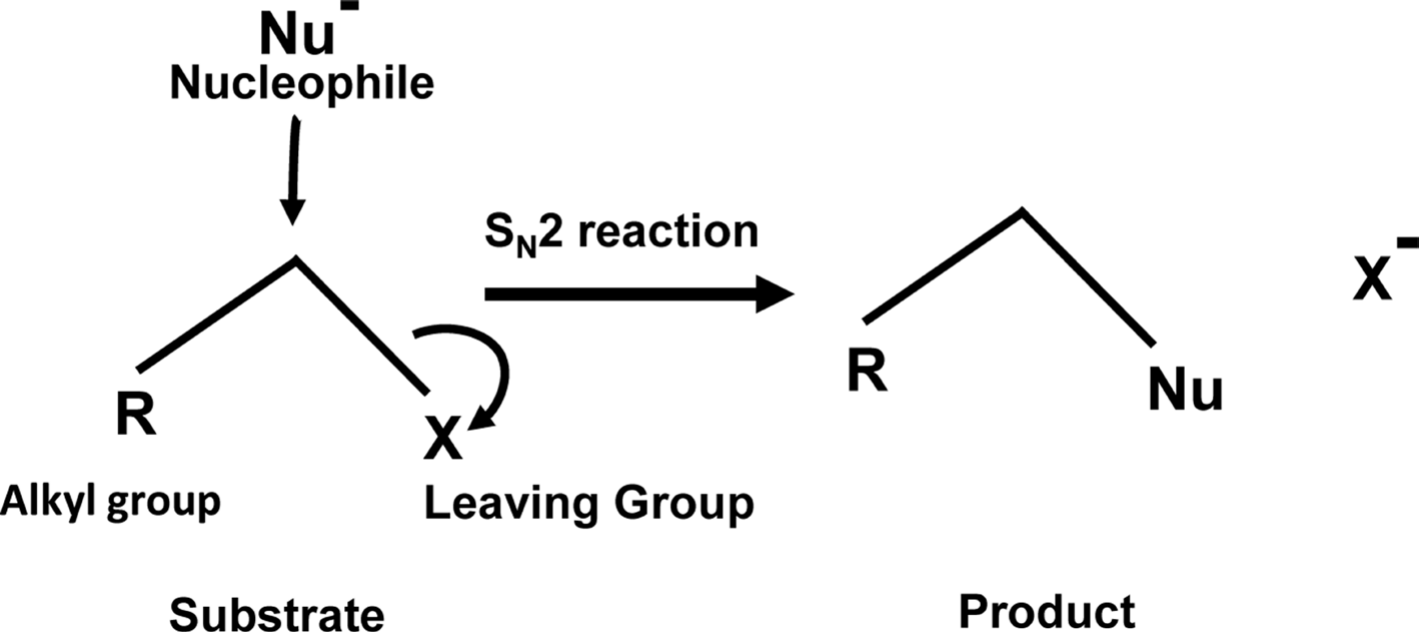
S_N_2 alkylation: Alkylation of the target by 3-BP follows the S_N_2 mechanism.

**Figure 4:**
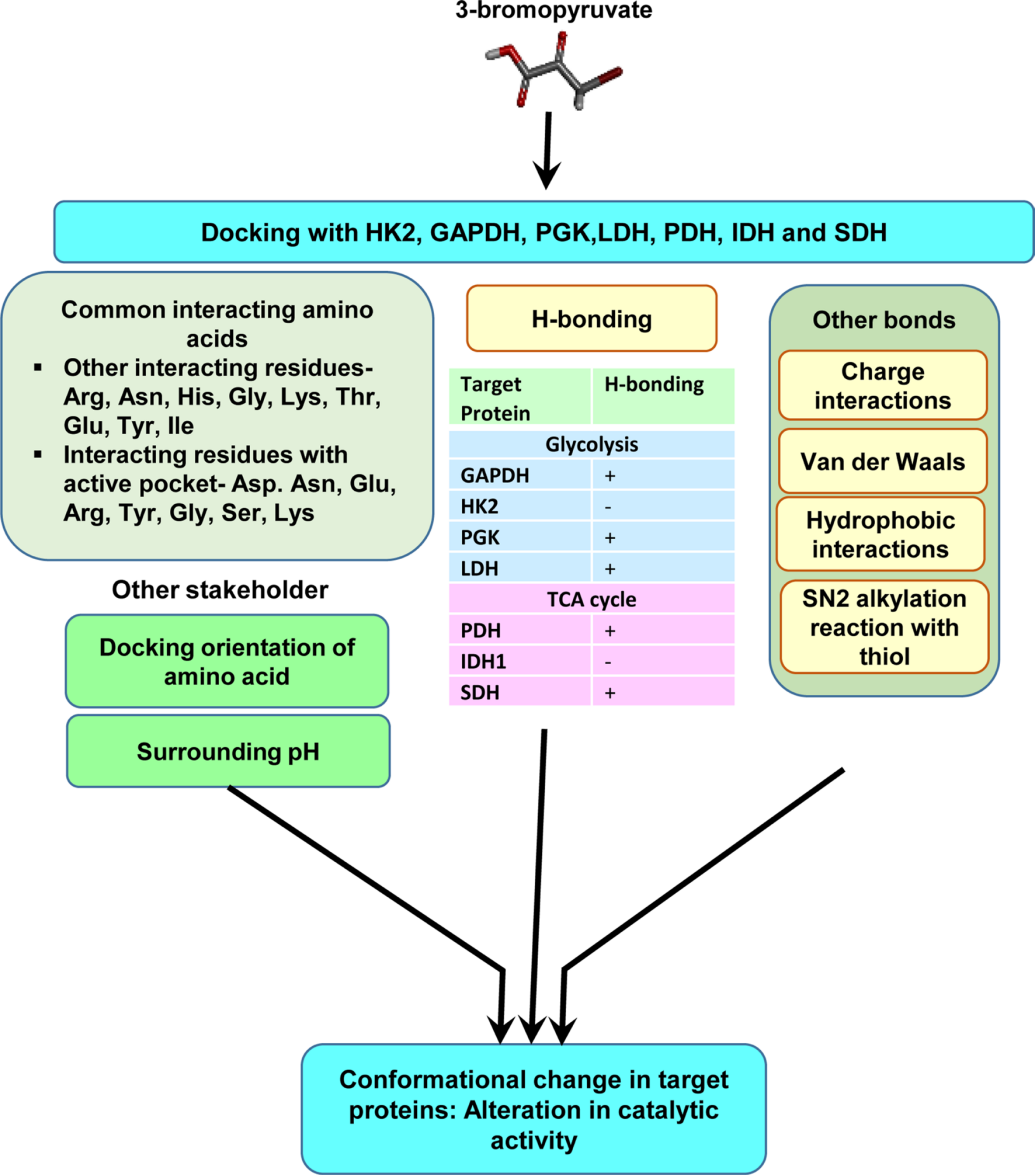
Molecular characterization of the interaction of 3-BP with multiple target molecules. Molecular docking studies indicate that, in addition to alkylation by S_N_2 reaction, 3-BP may impart modulatory actions on targets* via *multiple interactions including H-bonds, charged, and hydrophobic interactions. The diagram depicts interacting amino acids of the target molecules involved in docking. The nature of interactions could depend on docking orientations, the composition of active sites, and other environmental parameters, such as pH.

Given the diverse stakeholders of tumor metabolism, it is essential to evolve a multifaceted targeting strategy for effective control of neoplastic cells’ survival, invasion, and metastasis. Our survey of literature for drugs fulfilling the objective of targeting multiple aspects of tumor metabolism has fetched a promising hope from an agent, which is a brominated derivative of pyruvate, designated as 3-BP. It has shown tremendous antineoplastic potential with several merits over other metabolic inhibitors (Ko et al., 2001; Azevedo-Silva et al., 2016). The following section highlights the broad spectrum of the antineoplastic actions of 3-BP, along with the possible underlying mechanisms. Future possibilities for its applications in anticancer regimens are also discussed. Interestingly, 3-BP is capable of inhibiting several aspects of tumor metabolism related to nutrient uptake, rewired metabolic pathways, pH homeostasis, and metabolism-dependent cellular interactions in the TME. Being an alkylating agent, 3-BP targets a plethora of biomolecules of neoplastic cells (Azevedo-Silva et al., 2016; Lis et al., 2016). Moreover, 3-BP shows a high degree of specificity for its anticancer activity (Buijs et al., 2013; Ganapathy-Kanniappan et al., 2013). The tumor cell-specific selectivity of 3-BP mainly depends on the similarity of this molecule with lactate and pyruvate, and hence it utilizes common transporters to gain cellular entry. On the contrary, other alkylating agents mainly enter by diffusion across the plasma membrane and, therefore, lack specificity (Ganapathy-Kanniappan et al., 2013). MCT-1 and MCT-4, which are specifically upregulated in neoplastic cells, mediate transport of 3-BP (Queirós et al., 2012; Baltazar, 2014; Counillon et al., 2016). Nevertheless, the acidic environment of the tumor milieu proves thermodynamically favorable for the uptake of 3-BP by cancer cells because of the pH difference across the plasma membrane (Azevedo-Silva et al., 2015). Another important reason for its selectivity against neoplastic cells is caused by the unmatched ability of 3-BP to alkylate and inactivate metabolic enzymes, which are selectively upregulated in malignant cells (Chen et al., 2009; Ganapathy-Kanniappan et al., 2009; Gandham et al., 2015; Azevedo-Silva et al., 2016; Jardim-Messeder and Moreira-Pacheco, 2016). Being an electrophile, 3-BP covalently and irreversibly modifies its targets’ nucleophilic moieties *via* S_N_2 mechanism of alkylation (Azevedo-Silva et al., 2016; Oronsky et al., 2012) (**Figure 3**). Interestingly, it is also reported that 3-BP is a prodrug, which gets activated in the vicinity of target nucleophiles only (Oronsky et al., 2012). As shown in **Figure 4**, 3-BP is capable of alkylating and modifying several target enzymes of glycolysis and the TCA cycle and consequently is highly capable of reversing the Warburg effect in neoplastic cells, leading to induction of cell death (Lis et al., 2016). Critical metabolic enzymes reported to be inhibited by 3-BP include hexokinase 2 (HK2) and glyceraldehyde 3-phosphate dehydrogenase (GAPDH) in the glycolytic pathway; LDH and PDH in the linker pathway to TCA cycle; and SDH, isocitrate dehydrogenase, and α-ketoglutarate dehydrogenase in the TCA cycle (Chen et al., 2009; Ganapathy-Kanniappan et al., 2009; Ganapathy-Kanniappan et al., 2013; Azevedo-Silva et al., 2015; Jardim-Messeder and Moreira-Pacheco, 2016; Yadav et al., 2017b). Moreover, many other targets have been identified, including V-ATPase, pyruvate kinase, ribonuclease A, and glutathione (Dell’Antone, 2006; Ganapathy-Kanniappan et al., 2009; Ehrke et al., 2015). Nevertheless, 3-BP can alkylate several amino acids, particularly the cysteine moieties in several proteins (Hanau et al., 1995; Ganapathy-Kanniappan et al., 2009). Additionally, 3-BP also reported inhibiting glyoxalase and serine hydroxyl ethyl transferase (Valenti et al., 2015; Paiardini et al., 2016). Given the broad spectrum of the inhibitory action of 3-BP on metabolic enzymes, it is capable of ushering a “metabolic catastrophe” in cancer cells, leading to the depletion of ATP generation, causing declined neoplastic cell survival (Davidescu et al., 2015; Sun et al., 2015). Because of the massive antimetabolic potential, an ever-increasing list of cancer targets is building up, which display susceptibility to the antineoplastic action of 3-BP. Cytotoxic action of 3-BP is exerted against neoplastic cells and animal tumor models of diverse origins such as breast, prostate, pancreas, cervix, kidney, colon, hematological, and pulmonary (Xu et al., 2005; Cao et al., 2008; Hulleman et al., 2009; Schaefer et al., 2012; Attia et al., 2015; Nilsson et al., 2015; Sun et al., 2015; Valenti et al., 2015; Azevedo-Silva et al., 2016). Recently, our group reported a strong antitumor action of 3-BP against tumor cells of thymic origin, which are one of the rarest cancers and hence difficult for therapeutic exploration (Yadav et al., 2017a; Yadav et al., 2017b). In addition to its ability to inhibit metabolic enzymes, 3-BP also causes overexpression of the reactive oxygen species (ROS), along with depletion of ROS scavenger glutathione (GSH), in neoplastic cells, which in turn can induce cell death by induction of apoptosis and necrosis (Kim et al., 2008; Valenti et al., 2015). Nevertheless, ROS increases cellular and endoplasmic reticular (ER) stress (Ganapathy-Kanniappan et al., 2010). Moreover, reports suggest that, in addition to ER stress, 3-BP can contribute to the inhibition of protein synthesis (Kwiatkowska et al., 2016). Such perturbations are also associated with unfavorable modulation of redox homeostasis, accompanying mitochondrial damage (Kwiatkowska et al., 2016; Lis et al., 2016). Additionally, 3-BP treatment leads to the release of VADC-associated HK2, diminished mitochondrial potential, the release of cytochrome *c*, downregulation of antiapoptotic Bcl-2 and Mcl-1, and activation of caspase-3, indicating the mitochondrial mode of apoptosis (Chen et al., 2009; Yadav et al., 2017a; Yadav et al., 2017b).

Furthermore, it is demonstrated that 3-BP can render tumor cells susceptible to the induction of cell death by additional mechanisms, including modulated expression of HIF-1α and by modulating pH homeostasis associated with altered glycolysis and TCA cycle (Marín-Hernández et al., 2009; Semenza, 2010). As already elaborated, HIF-1α can alter carbohydrate metabolism, oxidative stress, expression of cell survival-modulating cytokines, and mediators of drug resistance, enhancing chemosensitivity of tumor cells (Semenza, 2010; Masoud and Li, 2015). Nevertheless, 3-BP-dependent alterations in HIF-1α expression can lead to a declined expression of GLUT-1 and consequently glucose uptake by neoplastic cells, interfering with the lifeline of the nutrition supply of neoplastic cells (Orue et al., 2016; Yadav et al., 2017b). 3-BP-dependent inhibition of MCT-1 expression and, hence, lactate transport can cause a rise in intracellular pH accompanied by altered tumor acidosis (Sun et al., 2015; Yadav et al., 2017b). *In vivo* study in the murine tumor-bearing model has demonstrated 3-BP-dependent reconstitution of the cellular and soluble component of the TME. The TME of 3-BP-administered tumor-bearing hosts displayed repolarization of macrophages to tumoricidal M1 phenotype, accompanied by an increase in CD4, CD8, CD49, CD25 (IL-2R), and CD62L, CD11c, and TLR-4 expressing cells, indicating an altered repertoire of NK cells and T lymphocytes in the TME and alleviation of tumor-associated immunosuppression (Yadav et al., 2017a). Interestingly, it was demonstrated that 3-BP administration could inhibit the expression of V-ATPase in tumor cells, further contributing to the deregulation of pH homeostasis (Dell’Antone, 2006). The 3-BP-dependent altered internal milieu in tumor cells could also be linked to the decline of FASN expression, which is suggestive of inhibited *de novo* fatty acid synthesis, necessary for membrane biogenesis (Yadav et al., 2017a). Additionally, 3-BP can lead to a decline in HSP70 expression, suggesting the declined ability of 3-BP-exposed tumor cells to cope with stressful conditions, rendering them susceptible to induction of cell death (Yadav et al., 2017a). Indeed, other workers have indicated an increase in stress markers in 3-BP-treated cancer cells (Ganapathy-Kanniappan et al., 2010; Chiasserini et al., 2017). A decline of the stress-bearing capacity can augment cell death and the expression of VEGF, which triggers a diminished vasculature and blood flow in the TME (Attia et al., 2015; Yadav et al., 2017a). The 3-BP-dependent cell cycle arrest in tumor cells can be yet another trigger leading to induction of cell death (Chong et al., 2017; Yadav et al., 2017a). Additionally, 3-BP has been shown to interfere with oxidative phosphorylation (Lis et al., 2016). Furthermore, 3-BP targets complexes I and II of ETC, which also contributes to ATP depletion (Jardim-Messeder and Moreira-Pacheco, 2016).

**Figure 5:**
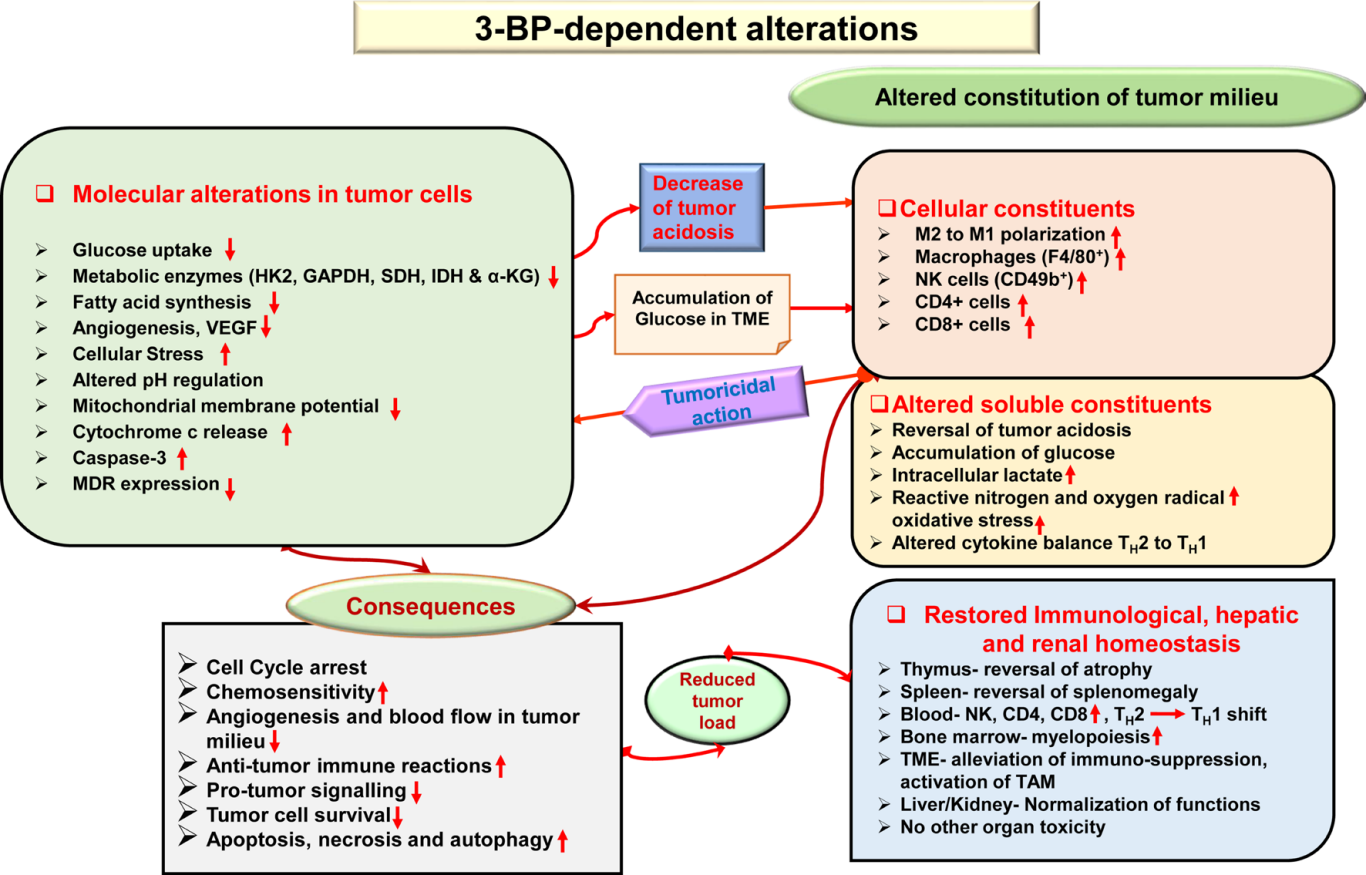
Multifaceted antineoplastic actions of 3-BP. Molecular mechanisms of the antineoplastic action of 3-BP involve multifaceted targeting of critical molecules involved in tumor metabolism leading to a metabolic catastrophe in neoplastic cells. A broad spectrum of antitumor actions can be manifested by 3-BP, which included the generation of ER and mitochondrial stress, inhibition in the expression of multidrug resistance (MDR) molecules, altered pH and redox homeostasis, depleted glucose uptake, and reconstitution of cellular, soluble, and biophysical components of the TME. Many of these actions are direct, whereas others could be manifested indirectly* via *other molecules such as altered cytokine balance and ROS. The antitumor action of 3-BP implicates cell cycle arrest, altered chemosensitivity, a decline of blood supply in the TME, inhibition of immune evasion, immune augmentation, and a decline of protumor signaling. Consequently, because of multiple effects and depletion of energy generation, tumor cells undergo cell death* via *induction of apoptosis, necrosis, and autophagy. Recuperative effect of 3-BP is imparted on the function of vital organs, such as the liver and kidney. Abbreviations: Cyc c, cytochrome *c*; pHi, intercellular pH; V-ATPase, vascular-ATPase; VEGF, vascular endothelial growth factor.

Given the fact that most anticancer drugs inflict cytotoxicity to normal cells, tissues, and organs in cancer patients (Cheok, 2012), safety concerns are of primary focus while designing and developing chemotherapeutic agents. It has been demonstrated that the antitumor action of 3-BP is accompanied by protective and recuperative effects on immunological, hepatic, and renal homeostasis, with normalization of liver and kidney functions, reduction of tumor growth-associated splenomegaly, restored thymic homeostasis, normalization of blood lymphocytes, and upregulated myelopoiesis (Yadav et al., 2018). Additionally, other studies showed that 3-BP was safe to various tissues (Kunjithapatham et al., 2013; Pan et al., 2016), displaying minimal hepatic and nephrotoxicity (Pan et al., 2016). In **Figure 5**, a summary of novel antitumor mechanisms of 3-BP is depicted, showing its ability of multifaceted antitumor action, encompassing aspects such as membrane transport, inhibiting metabolic pathways, pH homeostasis, reconstitution of the TME, declined lipid biosynthesis, mitochondrial stress, restored organ homeostasis, and chemosensitivity.

Most of the antineoplastic actions of 3-BP are mainly attributed to its ability to alkylate a variety of target molecules in neoplastic cells. However, because of the lacuna concerning the biochemical mechanism(s) of its binding to various heterogeneous target molecules, we carried out an extensive *in silico*-based investigation to precisely understand the molecular nature of the binding of 3-BP to its target proteins of glycolysis and TCA cycle (Yadav et al., 2017c). Docking analysis of 3-BP was carried out against the most vulnerable targets, namely, HK2, GAPDH, LDH, SDH, PDH, phosphoglycerate kinase (PGK), and IDH1 enzymes of carbohydrate metabolism (**Figure 4**) (Yadav et al., 2017c). Interestingly, this study demonstrated the implication of H-bonding between 3-BP and its targets, except for HK2 and IDH1. Moreover, Arg, Asn, Gly, His, Ser, and Thr were suggested to determine the binding strength between 3-BP and its target enzymes involving active sites (Yadav et al., 2017c). Another study has demonstrated the binding of 3-BP with one or more amino acids at the active site of the target enzymes (Silverman and Eoyang, 1987). Based on the calculation of geometric shape complementarity score, approximate interface, binding energy, and dissociation constant of the docking of 3-BP with target enzymes, it was demonstrated that 3-BP shows a stable binding to its targets (Yadav et al., 2017c). Furthermore, it was indicated that HK2, PDH, and SDH were the most preferred targets over the other enzymes. In addition to the H-bonds, other prominent biochemical interactions included hydrophobic interaction and Van der Waals forces, which vary by the amino acids of the respective docking sites (Pan et al., 2013; Yadav et al., 2017c).

Based on the ability to inhibit target enzymes, 3-BP derivatives have been tested for their antineoplastic activity. A derivative of 3-BP, named the 3-bromo-2-oxopropionate-1-propyl ester (3-BrOP), acts similarly as the 3-BP prodrug but was reported to be more stable than 3-BP and possessed a superior ability to deplete ATP in neoplastic cells (Lis et al., 2016). Similarly, we compared the docking ability of 3-BP derivatives dibromopyruvate (DBPA) and propionic acid (PA) with 3-BP target enzymes. Interestingly, DBPA was found to display a better docking ability than 3-BP and PA to various target enzymes (Yadav et al., 2017c), indicating strong antineoplastic potential, which needs to be explored further. These studies will also aid in optimizing the therapeutic efficacy of 3-BP by achieving a better understanding of the inhibition of target enzymes by modification of the catalytic site. In addition to the protective and recuperative actions of 3-BP in a tumor-bearing host, it is noteworthy that 3-BP and its derivatives DBPA and PA have been predicted for drug-likeness criteria and found to satisfactorily pass the parameters of drug-likeness on Lipinski filter and FAFDrugs 3 analysis tools (Yadav et al., 2017c).

## Limitations and Prospects

Despite approval for phase I trial by the FDA, the clinical trials with 3-BP have not yet been realized because of 1) limitation of financial resources for executing the trials and 2) death of three patients being attributed to 3-BP. However, it was later on reported that these deaths were not likely caused by 3-BP (Lis et al., 2016). Moreover, these controversies associated with the lethality of 3-BP when used in inappropriate dose regimens (Feldwisch-Drentrup, 2016) need to be addressed adequately under proper scientifically validated and controlled settings before its applications for human use as an anticancer drug. It is essential to consider physiological and physical parameters capable of influencing the antitumor efficacy of 3-BP. Despite this, isolated sporadic clinical applications of 3-BP in humans and xenograft models of human cancers have raised positive optimism for its use as a potent cancer therapeutic agent (Lee et al., 2017a). A study using a volunteer cancer patient demonstrated fruitful outcomes for the use of 3-BP as an anticancer drug (El Sayed et al., 2014). Moreover, these limited clinical trials have shown minimal side effects, except minor concerns regarding burning sensation and phlebitis (El Sayed, 2018). Most preclinical and clinical trials report about its safety concerning hepatic functions (Ko et al., 2012; El Sayed et al., 2014; Pan et al., 2016; Yadav et al., 2018). The main recommendations for overcoming the limitations regarding the use of 3-BP include strict implementation of only formulated preparations in human applications (El Sayed, 2018; Fan et al., 2019b), use of GSH scavengers accompanying 3-BP administration because GSH can inactivate 3-BP (El Sayed, 2018), and strict monitoring of the dose regimens (El Sayed, 2018). Furthermore, approaches of liposome-encapsulated 3-BP formulations are suggested to improve its adequate concentrations in the tumor milieu (Gandham et al., 2015). Use of 3-BP as an adjunct agent along with other conventional approaches is considered to hold therapeutic potential (Ganapathy-Kanniappan et al., 2013; Zhang et al., 2015; Chong et al., 2017).

There is a promising potential for 3-BP and its derivatives for being assessed further in preclinical and clinical settings to predict about anticancer efficacy. Critical checkpoints of tumor metabolism right from the level of import of nutrients through their metabolic channelization and generation of ATP are affected by 3-BP. Further, cellular signaling and pH homeostasis are also influenced by 3-BP and hence it is a highly capable agent for modulating metabolic linking in the TME. Thus, 3-BP displays multifaceted antineoplastic activity *via* its direct inhibitory action on metabolic targets of neoplastic cells, by harnessing the antitumor potential of the immune system, and by rendering the TME unfavorable for tumor progression. Hence, 3-BP ushers a promising hope in the combat against cancer because of its low cost, a broad spectrum of antineoplastic potential, desirable drugability characteristics, and a track record of safety, necessitating initiation of further clinical optimization.

## Author Contributions

SY: conceiving of the idea; a survey of the literature, writing, and preparation of the manuscript; SS: conceiving of the idea and manuscript preparation; SP, YG, and MT: writing manuscript.

## Conflict of Interest Statement

The authors declare that the research was conducted in the absence of any commercial or financial relationships that could be construed as a potential conflict of interest.
